# Bayesian Spatial Analysis of Trends and Disparities in Telehealth Use During the COVID-19 Pandemic: Retrospective Observational Study

**DOI:** 10.2196/73271

**Published:** 2026-01-20

**Authors:** Jong Hyung Lee, Roy T Sabo, Jacqueline Britz, Benjamin Webel, Kelly M Rodriguez, Evan French, Alex H Krist

**Affiliations:** 1 Department of Family Medicine and Population Health School of Medicine Virginia Commonwealth University Richmond, VA United States; 2 Department of Biostatistics School of Public Health Virginia Commonwealth University Richmond, VA United States; 3 Broadlands Family Practice/Inova Ashburn, VA United States; 4 C Kenneth and Dianne Wright Center for Clinical and Translational Research Office of the Vice President for Research and Innovation Virginia Commonwealth University Richmond, VA United States

**Keywords:** telehealth, COVID-19, health disparities, rural health, Bayesian analysis, telehealth use over time, Virginia

## Abstract

**Background:**

Telehealth has emerged as an essential health care tool, particularly during the COVID-19 pandemic, when in-person medical services were significantly restricted. While telehealth adoption surged during the pandemic, disparities in its access and use have been observed, especially among vulnerable populations. Understanding these trends and identifying barriers is crucial for promoting equitable health care delivery.

**Objective:**

This study aims to assess disparities in telehealth use across Virginia, focusing on demographic, socioeconomic, and geographic factors influencing access. Using spatial modeling, we evaluate the association between community-level characteristics and telehealth use. Our findings can highlight areas where telehealth remains underused, informing targeted interventions to improve equitable access.

**Methods:**

A retrospective observational analysis was conducted from 2016 to 2021 using data from the Virginia All-Payer Claims Database (APCD) and demographic data from the American Community Survey. Annual telehealth use rates were calculated at the zip code tabulation area level during the study period. Demographic and socioeconomic variables, such as educational attainment, poverty, and broadband internet access, along with geographic factors, including population density and rurality, were incorporated. A Bayesian spatial regression with conditional autoregressive priors on zip code tabulation area–level random effects was used to assess the relationship between telehealth use and community-level characteristics. The deviance information criterion was used to select the final model. Results were presented as relative risks (RRs) with 95% credible intervals.

**Results:**

The trends showed an increase in telehealth use during the pandemic, with rural areas showing the most notable rise in 2020 (41.2% of all the visits), up from 14.2% in 2016, representing a statistically significant upward trend (*P*<.001). However, by 2021, telehealth use shifted, with suburban areas leading (43.1% of the visits), while rural areas followed (37.7%), indicating evolving patterns of adoption over time. Some sociodemographic factors exhibited temporal shifts in their association with telehealth use. Disparities in telehealth use among older adults improved, as the adjusted RR increased from 0.74 in 2019 to 0.95 in 2020, though a slight decline was observed in 2021 (RR 0.92, 95% credible interval 0.89-0.96). Conversely, disparities among non-Hispanic Black populations widened, with adjusted RR declining from 0.96 in 2020 to 0.93 in 2021 (95% credible interval 0.90-0.97), signaling persistent disparities. Higher telehealth use was associated with better broadband access (adjusted RR 1.06, 95% credible interval 1.01-1.11) and increased population density (adjusted RR 1.07, 95% credible interval 1.02-1.12).

**Conclusions:**

Telehealth use surged in Virginia during the COVID-19 pandemic, particularly in rural areas. However, the findings indicate that disparities persist in the post–COVID-19 pandemic period, especially among minority population groups and older adults. Addressing these gaps requires targeted interventions, including expanding broadband infrastructure and improving telehealth literacy. These efforts are crucial to ensuring equitable access to telehealth services, especially for underserved communities.

## Introduction

### Background

Telehealth has slowly gained popularity in recent years, becoming a vital tool during and after the COVID-19 pandemic [1]. Before the COVID-19 pandemic, telehealth was primarily used in hospital settings and for ambulatory purposes, particularly in outpatient care for mental health, although at a significantly lower rate than in-person care [2,3]. Its broader adoption had been limited by technological barriers, concerns about reimbursement, and questions regarding maintenance of care standards [4,5]. However, the COVID-19 pandemic acted as a catalyst, forcing health care systems worldwide to adopt telehealth to ensure continued access to health care during lockdowns and publicly mandated social distancing measures [6,7].

### Prior Work

A major benefit of telehealth is its ability to increase access to care, particularly for populations with travel limitations or those in rural or underserved areas [8-10]. While clinicians recognize this benefit, they also note the challenges associated with telehealth, such as maintaining the quality of patient interactions, technical difficulties with internet connectivity, concerns over reimbursement structures, and the need for adequate training for both clinicians and patients [11].

Telehealth use in the United States increased substantially beginning in March 2020, peaking in April 2020, before gradually stabilizing [12,13]. National data indicate a significant increase in telehealth visits across all payer types during this period, with Medicare demonstrating the most substantial adoption growth [13]. This surge underscores the critical role of telehealth in maintaining health care access, particularly for high-risk populations, such as older adults, during the public health crisis. Recent studies report significant increases in telehealth use during the COVID-19 pandemic, followed by partial stabilization and variation according to payer type and geographic region [12,13]. Persistent inequities continue to harm rural and socioeconomically disadvantaged communities, where limited broadband, lack of appropriate devices, and gaps in digital literacy restrict access to services [8-11].

Usability and access barriers continue to limit effective patient engagement. However, interventions and solutions, such as simplified digital platforms, language interpretation services, and internet connectivity support, have demonstrated potential to mitigate these disparities [14,15]. In addition, audio-only telehealth encounters have improved convenience and sustained access for those lacking reliable video or broadband resources [16]. These findings highlight the importance of maintaining flexible telehealth modalities to ensure equitable health care.

### Regional Context

In the Commonwealth of Virginia, telehealth adoption mirrored national trends but displayed noticeable disparities in use across different payer types and regions. Preliminary analyses reveal that Medicaid beneficiaries lag behind Medicare enrollees in terms of telehealth use, suggesting that barriers to access may be more pronounced among the Medicaid population [13,17]. Many of the previous studies rely on short observation windows concentrated in early 2020 and coarse geographic units. Community-level socioeconomic factors are inconsistently used, and spatial dependence is often not incorporated in a model, risking biased estimates and masking local disparities. Understanding these disparities and identifying factors influencing telehealth adoption in Virginia are essential for developing targeted interventions to ensure equitable health care access.

### Objectives

The primary objective of this study is to evaluate telehealth use trends over time (particularly around the COVID-19 pandemic), identify demographic and socioeconomic factors contributing to disparities in access, and assess how geographic- and community-level characteristics influence telehealth adoption. In addition, this study aims to identify areas in Virginia where telehealth services remain underused, with the goal of informing future policy decisions and health care interventions.

## Methods

### Study Design and Data Sources

This retrospective observational study examined telehealth use in Virginia from 2016 to 2021, using 2 primary data sources. The first source was the Virginia All-Payer Claims Database (APCD), which provides comprehensive health care use data across all payer types, including private insurers, Medicare, and Medicaid. The APCD contains detailed information on health care services rendered, including telehealth visits. The second data source was the American Community Survey (ACS), which provides demographic and socioeconomic data at the zip code tabulation area (ZCTA) level. The ACS dataset included indicators of social determinants of health, such as educational attainment, poverty levels, broadband internet access, and population density. In addition, the Social Deprivation Index (SDI), developed by the Robert Graham Center, was used to assess the levels of social deprivation across communities [18].

### Rationale for Study Setting

Virginia provides an analytically rich context for examining telehealth use because it captures a full spectrum of access environments—urban, suburban, and rural—including Northern Virginia and Appalachian localities. Documented broadband gaps, especially in rural communities, create meaningful variation in digital infrastructure that directly bears on telehealth use. Finally, this study leverages unique access to the Virginia APCD, which aggregates paid claims from commercial insurers, Medicaid, and Medicare, thereby enabling robust small-area inference that is not feasible in many other settings.

### Study Population

The study population consisted of Virginia residents who used both in-person and telehealth services between January 1, 2016, and December 31, 2021. Individuals were included if they had at least 1 claim in the APCD throughout the study period and had a valid ZCTA identifier. Participants with non-Virginia ZCTA (ie, nonresidents), incomplete records, and individuals with missing or invalid ZCTA information were excluded. The inclusion of ZCTA-level data allowed for the geographic analysis of telehealth use patterns and the identification of community-level factors influencing use.

### Variables and Measures

The primary outcome variable for this study was the telehealth use rate, defined as the proportion of unique patients per ZCTA who used telehealth services in a given year. This rate was computed for each year from 2016 to 2021 to observe temporal trends. Independent variables included demographic factors as well as socioeconomic indicators from the ACS, including educational attainment, broadband internet access, poverty status (measured as the proportion of the population below 200% of the federal poverty level), and population density. Patient-level usability metrics, such as task success and time to connect, were not directly measured in this study. Use differences were interpreted as population-level indicators reflecting convenience and access constraints.

For geographic factors, 2 approaches were used. First, rurality was classified using the ZCTA local assignments from the National Center for Education Statistics. This classification distinguishes between urban, suburban, and rural areas and is commonly applied in education and geographic research [19]. Second, for statistical modeling, population density was used as a continuous variable to account for geographic variation in telehealth use. The inclusion of broadband access as an independent variable was critical, as telehealth services rely heavily on reliable internet connectivity, especially in rural areas where broadband availability may be limited.

### Covariate Selection and Multicollinearity Assessment

Candidate covariates were specified based on previous literature and conceptual relevance to this study’s research questions. To minimize redundancy, variance inflation factors computed from an initial model using all candidate ZCTA-level predictors were examined. Variables with a variance inflation factor greater than 5.0 were considered collinear. In such cases, the variable demonstrating stronger construct validity and data completeness was retained. Some potential covariates, such as the Gini index, unemployment rate, the percentage of the population below 100% of the federal poverty level, and the percentage with a bachelor’s degree or higher, were excluded from the final model due to multicollinearity. The final model included only nonredundant covariates.

### Statistical Analysis

A Bayesian spatial regression model was used to estimate the relationship between telehealth use rates and community-level factors, accounting for spatial autocorrelation among ZCTAs. The Bayesian spatial model used a covariance structure proposed by Besag et al [20] to account for both spatially structured and unstructured variation across neighboring ZCTAs [20]. An adjacency matrix was created using queen contiguity [21], where contiguous ZCTAs were considered neighbors (ie, if they shared a common boundary). Spatial dependencies were given a conditional autoregressive prior, ensuring the model properly addressed spatial correlation in the data. The primary exposure variable was population density, while additional covariates included socioeconomic indicators, demographic characteristics, and health care access measures. These covariates were included in the fully adjusted model to assess their influence on telehealth use. Though telehealth data were summarized each year between 2016 and 2021, models were only built separately for the years 2019, 2020, and 2021. This decision reflected the substantial changes in telehealth patterns during these years, driven by evolving health care policies and patient behaviors, which make them the most relevant for understanding more recent use dynamics.

The number of telehealth visits per ZCTA was modeled using a Poisson distribution, with the log of the population size in each ZCTA included as an offset to account for differences in exposure. Both spatially structured and unstructured random effects were included to account for spatial heterogeneity and overdispersion. To efficiently estimate the posterior distributions of parameters and random effects, the integrated nested Laplace approximation (INLA) method was used, implemented through the *R-INLA* package [22]. This approach was favored for its computational efficiency and accuracy compared to the more time-intensive Markov chain Monte Carlo methods. Posterior distributions for each regression coefficient were approximated, and the relative risk (RR) with 95% credible intervals for each covariate was reported by exponentiating these mean estimates, facilitating statistical inference regarding the associations between telehealth use and community-level characteristics.

Model selection was guided by the deviance information criterion (DIC), with a lower DIC indicating better model fit. The models compared included the following:

- Model 1, namely, crude model, included only population density and accounted for unstructured (nonspatial) variation.
- Model 2, namely, crude spatial model, included only population density and accounted for both spatially structured correlation and unstructured (nonspatial) variation.
- Model 3, namely, fully-adjusted model, incorporated all covariates and accounted for both spatially structured correlation and unstructured (nonspatial) variation.

Exceedance probabilities were also computed to identify ZCTAs with a high probability of telehealth use rates falling below a specific threshold, enhancing the spatial assessment of underuse [23]. Since 2019 was a pre–COVID-19 pandemic year, the telehealth use rate across Virginia was significantly lower, necessitating a 10% threshold to effectively identify underuse patterns in that year. By contrast, a 50% threshold was used for 2020 and 2021 to capture disparities in access as telehealth use increased during the pandemic. All statistical analyses were conducted in RStudio (version 1.3.1093; Posit, PBC).

### Ethical Considerations

This study was reviewed by the Virginia Commonwealth University Institutional Review Board (IRB) and determined not to be human participant research. The original data collection was performed with IRB approval (HM20031637). The authors obtained permission to access and use the data. This study used only deidentified, aggregated data from the Virginia APCD and the ACS; therefore, it did not require additional IRB review. No identifiable patient information or sensitive personal data were used in this study, ensuring compliance with ethical standards for the use of deidentified data.

## Results

### Overview

**Figure 1:**
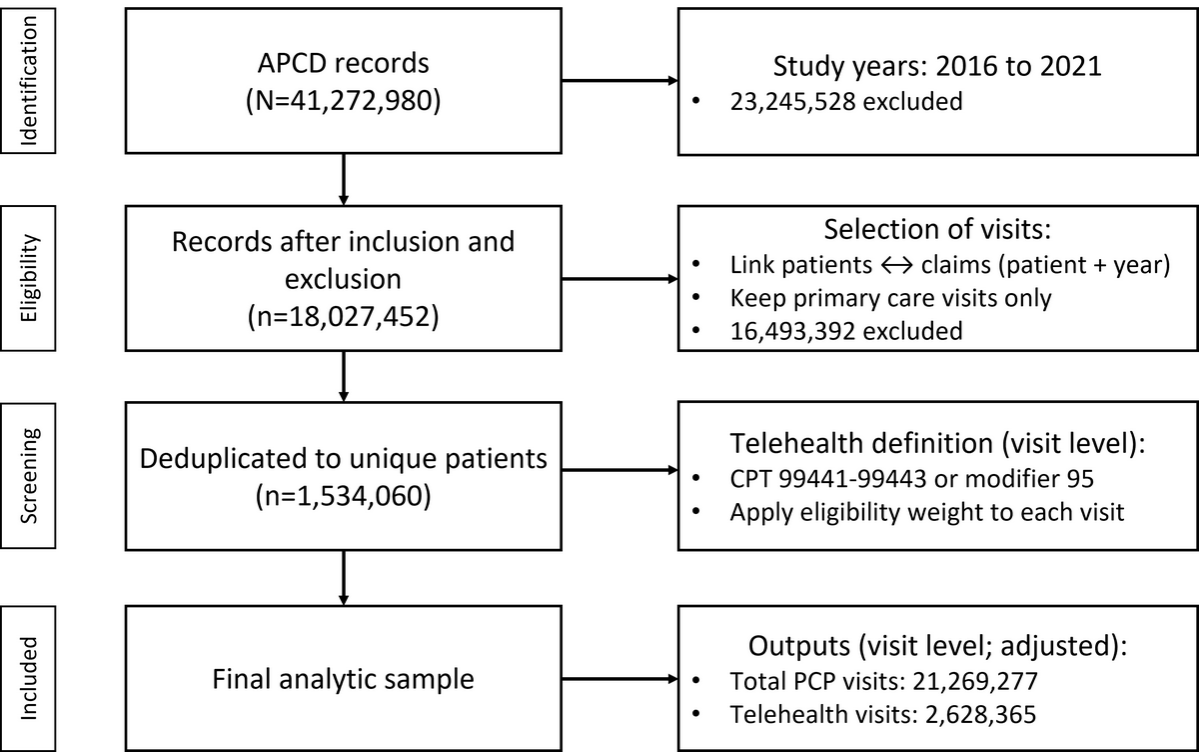
APCD cohort selection (2016-2021) and visit-level telehealth counts in primary care. APCD: All-Payer Claims Database; CPT: Current Procedural Terminology; PCP: Primary Care Physician.

The final analytic cohort comprised 1,534,060 unique patients. Figure 1 presents a sample selection flow diagram detailing the number of records at each stage of processing, from the initial APCD extraction through successive exclusions to the final analytic dataset.

### Temporal Variation in Telehealth Use

**Figure 2:**
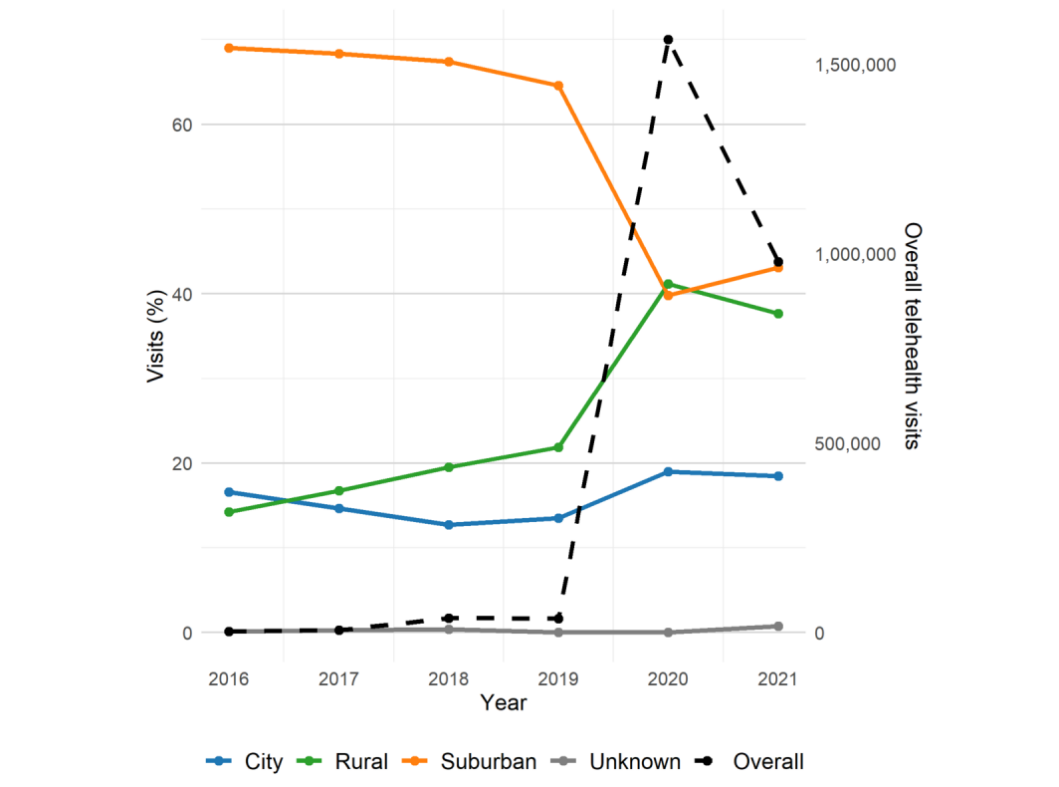
Temporal variation in telehealth use in primary care by rurality and year.

Our analysis of telehealth use rates from 2016 to 2021 revealed variations in adoption by rurality, year, and health care needs (Figure 2). In 2016, telehealth use was primarily concentrated in suburban or town areas, which accounted for 69% (2318/3359) of all telehealth visits. City areas followed with 16.6% (n=558) of the telehealth visits, while rural areas had the lowest telehealth use rate at 14.2% (n=478). These patterns persisted from 2016 to 2019. In early 2020, with the onset of the COVID-19 pandemic, telehealth use surged across all regions, with rural areas experiencing the most substantial increase, reaching 41.2% (644,404/1,564,817), representing a statistically significant upward trend (*P*<.001) (Figure 2).

While suburban areas also saw significant growth—from 23,980 visits in 2019 to 622,947 in 2020—this increase is not proportionally reflected in Figure 2, as the figure displays percentages relative to total visits per year rather than absolute counts. A closer examination of the data indicated that rural areas exhibited the largest relative increase in use during the COVID-19 pandemic. In contrast, urban and suburban areas continued to experience growth, although at a slower pace than rural areas.

Despite a slight decline in telehealth use across all regions in 2021, use levels remained elevated compared to pre–COVID-19 pandemic years. In 2021, suburban areas accounted for 43.1% (421,564/978,207) of the telehealth visits, rural areas for 37.7% (n=368,343) of the telehealth visits, and city areas for 18.5% (n=180,754) of the telehealth visits. Although city areas had a smaller share of total visits, differences in telehealth adoption rates across geographic areas are further explored subsequently.

### Bayesian Spatial Regression Findings

#### Model Comparison and Fit

The selection of the final model was based on the DIC, with lower values indicating better model fit:

- Model 1 included only population density and an unstructured (nonspatial) random effect (DIC=7956.42).
- Model 2 included only population density and both a spatially structured random effect and an unstructured random effect (DIC=7942.35).
- Model 3 included population density and all covariates, with both spatially structured and unstructured random effects (DIC=7939.77).

#### Community-Level Factors Affecting Telehealth Use

On the basis of the fully adjusted Bayesian spatial regression model, telehealth use was substantially associated with several community-level factors. As shown in Table 1, the proportion of individuals with disabilities was positively associated with higher telehealth use (adjusted RR 1.07, 95% credible interval 1.03-1.12). In addition, areas with higher population density were more likely to adopt telehealth (adjusted RR 1.07, 95% credible interval 1.02-1.12).

**Table 1:** Telehealth use rate (2019-2021) associations—Bayesian spatial analysis.

Zip code tabulation area–level factors^a^			2019				2020				2021		
			CRR^b^ (95% credible interval)^c^		ARR^d^ (95% credible interval)^e^		CRR (95% credible interval)^c^		ARR (95% credible interval)^e^		CRR (95% credible interval)^c^		ARR (95% credible interval)^e^
**Demographic (%)**													
	Hispanic	1.06 (0.96-1.16)		1.07 (0.95-1.22)		0.99 (0.97-1.01)		0.99 (0.98-1.02)		0.98 (0.95-1.02)		0.99 (0.96-1.02)	
	Non-Hispanic Black	0.96 (0.82-1.11)		0.97 (0.81-1.17)		0.94 (0.91-0.96)		0.96 (0.93-0.99)		0.93 (0.90-0.96)		0.93 (0.90-0.97)	
	Aged >65 y	0.84 (0.68-1.04)		0.74 (0.58-0.94)		0.97 (0.94-0.99)		0.95 (0.92-0.98)		0.95 (0.91-0.98)		0.92 (0.89-0.96)	
	Aged <5 y	1.08 (0.94-1.24)		1.13 (0.97-1.32)		1.01 (0.99-1.03)		1.01 (0.99-1.04)		1.03 (1.00-1.06)		1.04 (1.01-1.07)	
	Disability	1.22 (1.01-1.48)		1.61 (1.27-2.04)		1.00 (0.97-1.02)		1.03 (1.01-1.06)		1.00 (0.97-1.04)		1.07 (1.03-1.12)	
**Socioeconomic**													
	High school or less (%)	0.77 (0.63-0.94)		0.67 (0.51-0.87)		0.92 (0.89-0.95)		0.98 (0.94-1.02)		0.90 (0.86-0.94)		0.94 (0.89-0.99)	
	Below 200% federal poverty level (%)	0.97 (0.82-1.16)		0.84 (0.61-1.17)		0.95 (0.92-0.97)		0.99 (0.95-1.02)		0.96 (0.92-0.99)		1.01 (0.96-1.06)	
	Medicaid (%)	0.93 (0.80-1.07)		0.80 (0.63-1.01)		0.95 (0.93-0.97)		0.95 (0.93-0.98)		0.97 (0.94-0.99)		0.97 (0.93-1.01)	
	Social Deprivation Index	1.05 (0.94-1.18)		1.26 (1.03-1.55)		0.97 (0.95-0.99)		1.02 (0.99-1.06)		0.96 (0.93-0.99)		0.99 (0.95-1.03)	
**Access to care (%)**													
	No health insurance	1.03 (0.90-1.18)		1.01 (0.81-1.24)		0.96 (0.94-0.98)		0.97 (0.95-0.99)		0.97 (0.94-0.99)		0.97 (0.94-1.00)	
	Broadband internet	1.11 (0.82-1.51)		0.98 (0.69-1.39)		1.10 (1.05-1.15)		1.06 (1.01-1.11)		1.06 (0.99-1.14)		1.02 (0.95-1.09)	
**Geographic setting**													
	Population density	1.05 (0.92-1.21)		0.99 (0.85-1.14)		1.04 (1.01-1.07)		1.04 (1.01-1.07)		1.06 (1.01-1.11)		1.07 (1.02-1.12)	

^a^All zip code tabulation area–level factors were standardized.

^b^CRR: crude relative risk.

^c^Univariate model included each variable and structured and unstructured random effects.

^d^ARR: adjusted relative risk.

^e^Included population density as the primary exposure variable with all other covariates and structured and unstructured random effects.

#### Disparities in Telehealth Access

The analysis also revealed several disparities in telehealth use. The proportion of non-Hispanic Black residents in a ZCTA was negatively associated with telehealth use (adjusted RR 0.93, 95% credible interval 0.90-0.97). Similarly, older populations (aged >65 y) were less likely to use telehealth (adjusted RR 0.92, 95% credible interval 0.89-0.96).

#### Trends in Telehealth Use From 2019 to 2021

As presented in Table 1, spatial regression models for 2019 to 2021 provided a broader context for understanding how telehealth use evolved before, during, and after the pandemic. In 2020, broadband internet access was associated with higher telehealth adoption (adjusted RR 1.06, 95% credible interval 1.01-1.11). The proportion of non-Hispanic Black residents and older adults remained negatively associated with telehealth use, a trend that persisted into 2021.

In 2019, before the COVID-19 pandemic, telehealth use was more strongly associated with the SDI and the proportion of individuals with disabilities. The SDI, an index of area-level social disadvantage (higher values indicate greater deprivation), was positively associated with telehealth use (adjusted RR 1.26, 95% credible interval 1.03-1.55). Similarly, areas with a higher proportion of individuals with disabilities were more likely to use telehealth (adjusted RR 1.61, 95% credible interval 1.27-2.04). However, this association diminished in subsequent years, with the adjusted RR declining to 1.03 in 2020 and 1.07 in 2021. However, disparities among non-Hispanic Black populations persisted, with adjusted RR values declining from 0.96 in 2020 to 0.93 in 2021.

### Spatial Distribution of Telehealth Use

#### Geographic Disparities in Telehealth Use

**Figure 3:**
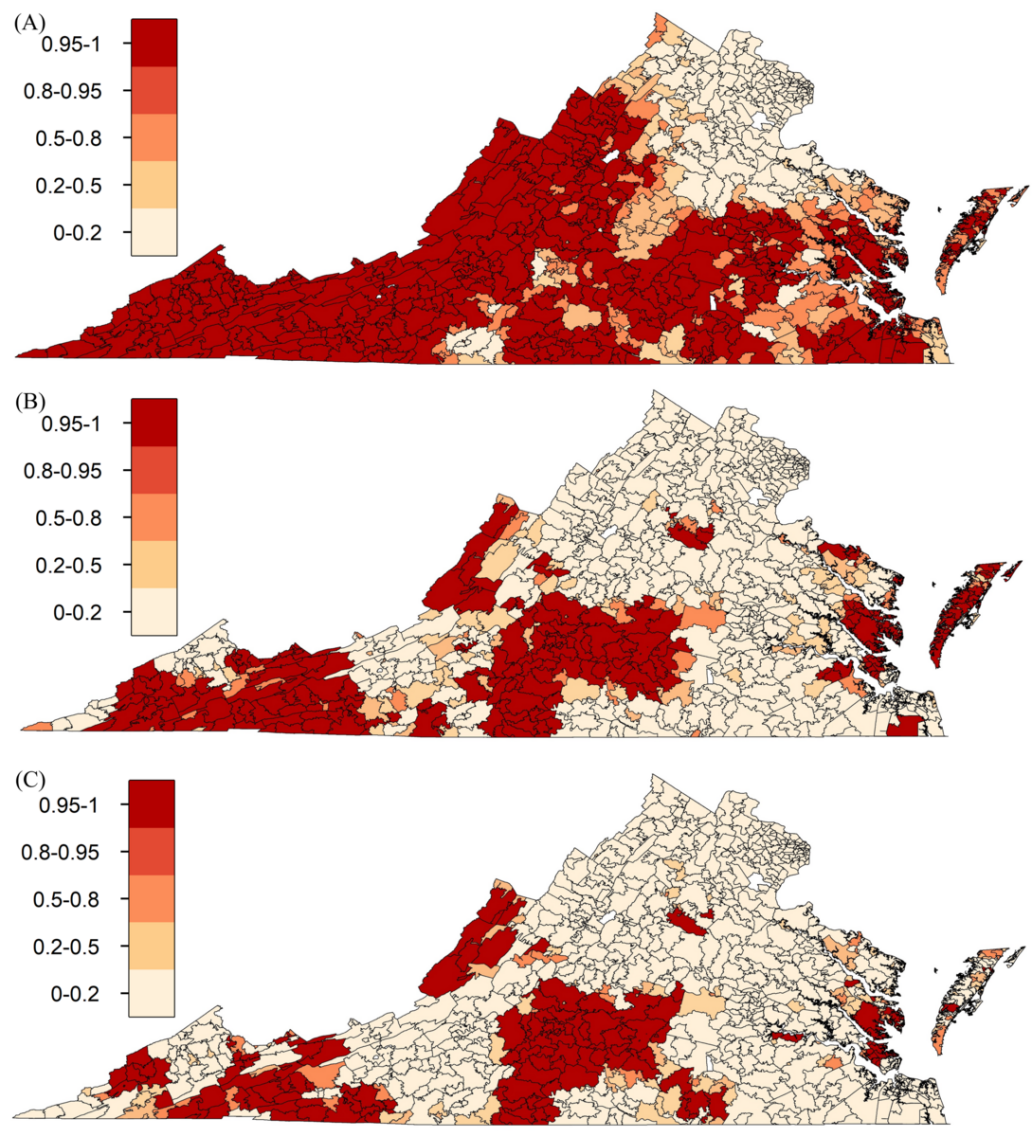
Probability of zip code tabulation areas (ZCTAs) exhibiting telehealth use rates below the Virginia average—10% in 2019 (A), 50% in 2020 (B), and 50% in 2021 (C).

Figure 3 illustrates the spatial distribution of telehealth use across Virginia, identifying several geographic areas with notably lower telehealth adoption. These regions included the Southwest, West Central, Southside, Valley, Hampton Roads, and Eastern regions, where ZCTAs exhibited telehealth use rates at least 50% lower than the state average.

#### Telehealth Use Disparities

Although Figure 3 illustrates ZCTAs with telehealth use rates below the Virginia average (10% in 2019 and 50% in 2020 and 2021), the analysis also identified some telehealth hot spots, mainly concentrated in Northern Virginia. Hot spots are defined as geographic clusters where telehealth use rates are significantly higher than the state average [24].

### Telehealth Use by Medical Condition

**Figure 4:**
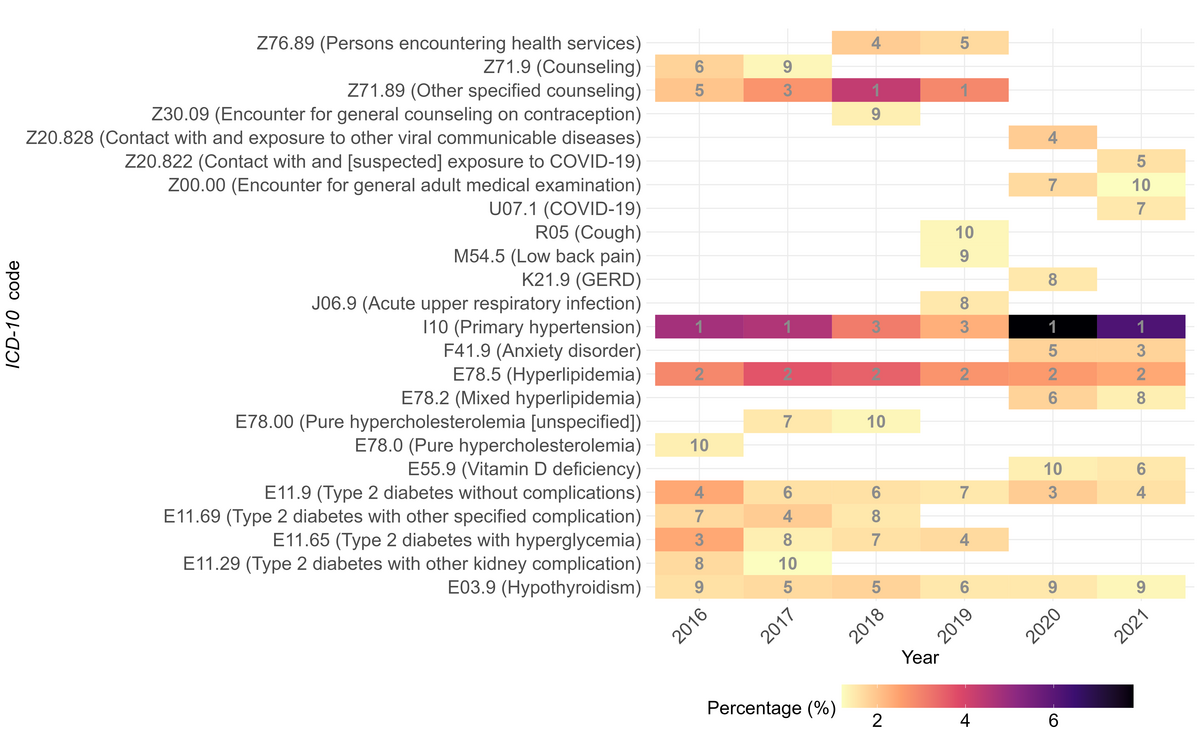
Heat map depicting telehealth visits for primary care’s top 10 International Classification of Diseases, 10th Revision (ICD-10) diagnoses in primary care, arranged by year (the gray numbers within each cell indicate the respective annual rankings). GERD: gastroesophageal reflux disease.

Figure 4 presents a heat map of telehealth visits for the top 10 *International Classification of Diseases, 10th Revision* diagnoses in primary care from 2016 to 2021. The data indicated that primary hypertension (I10) consistently ranked as one of the most common conditions for telehealth visits, particularly during the COVID-19 pandemic. This was followed by hyperlipidemia (E78.5) and type 2 diabetes mellitus without complications (E11.9), both of which also saw increases in telehealth visits during the pandemic. Mental health conditions, particularly anxiety disorder (F41.9), saw the most pronounced increase in telehealth visits during the COVID-19 pandemic. COVID-19–specific *International Classification of Diseases, 10th Revision* codes (ie, U07.1, Z20.828, and Z20.822) rose to the top 10 in 2020 and 2021.

## Discussion

### Principal Findings

This study provides a comprehensive analysis of telehealth use trends and disparities in Virginia during the COVID-19 pandemic. The findings reveal a significant increase in telehealth adoption starting in 2020, with use peaking during the height of the COVID-19 pandemic and remaining elevated compared to pre–COVID-19 pandemic levels. These results underscore the critical role telehealth played in maintaining health care access during a period of unprecedented disruption to traditional health care delivery [17]. The analysis identified key factors associated with telehealth use. Broadband internet access and population density were positively associated with higher telehealth use, highlighting the importance of digital infrastructure in facilitating access to telehealth services. However, broadband access did not exhibit a statistically significant association with telehealth use in either the pre–COVID-19 pandemic (2019) or post–COVID-19 pandemic (2021) periods.

Conversely, ZCTAs with higher proportions of the non-Hispanic Black residents, older adults, and Medicaid beneficiaries were negatively associated with telehealth use, highlighting disparities that may be driven by socioeconomic, technological, and systemic barriers. Telehealth has proven to be a valuable tool for expanding and broadening access to primary care services, particularly for managing chronic conditions, such as blood pressure monitoring and providing mental health care. Its integration into health care systems could improve access for populations with limited mobility or those residing in areas with health care provider shortages.

### Comparison With Existing Literature

The findings of this study are consistent with national trends that have documented increased telehealth adoption during the COVID-19 pandemic [25]. Studies have shown that the shift to telehealth was driven by the need to maintain health care access while minimizing in-person contact, and its adoption was facilitated by short-term policy changes, such as expanded reimbursement for telehealth services [26,27]. The disparities identified in this study align with existing literature on the digital divide, which has highlighted the challenges faced by underserved populations, including racial and ethnic minority groups, in accessing telehealth services [25,28-30].

### Reducing Telehealth Disparities Through Practice and Policy

Although telehealth is not suitable for every clinical condition, it is an essential tool for mitigating geographic and logistical barriers to health care access. Achieving equitable access requires coordinated efforts on infrastructure, financing, education, and inclusive design. Expanding broadband infrastructure and improving the affordability and reliability of internet service are crucial to equitable telehealth access. Targeted internet connectivity improvements in rural and low-income communities, coupled with public-private partnerships that establish telehealth access points in locations such as clinics, can improve availability and consistency of health care services [31,32]. These strategies are particularly important in areas where inadequate digital infrastructure overlaps with elevated rates of chronic disease and limited health care resources.

Equitable telehealth delivery depends on robust reimbursement and incentive policies. Payment parity policies for in-person and telehealth services can encourage broader adoption among health care providers, including those serving Medicaid enrollees and other low-income populations [30,33]. Enhanced funding for telehealth initiatives in medically underserved areas, particularly those characterized by poverty and lower educational attainment, is crucial for sustaining equitable service delivery [34].

Improving digital and telehealth literacy across populations with historically limited access is essential. Education and training programs that are accessible and tailored to older adults, racial and ethnic minority groups, and individuals with limited digital proficiency can reduce the digital divide [30,32,35]. Programs or initiatives that engage caregivers, community organizations, and peer-support networks can further increase user confidence in using telehealth platforms. Prioritizing inclusive design and accessibility is needed for equitable telehealth implementation. Telehealth platforms should incorporate features such as screen-reader compatibility, captioning, language interpretation services, and low-bandwidth modes to support users with disabilities or limited digital resources [29,35,36]. Device loan programs and mobile-friendly interfaces can increase participation among populations with limited access to technology.

Community engagement is critical for fostering trust and promoting digital inclusion and achieving digital equity. Collaborations with established local organizations can also facilitate outreach, raise awareness, and address cultural barriers, particularly among historically marginalized groups [34,36]. These partnerships can establish feedback mechanisms to ensure telehealth systems adapt to evolving community needs.

Ongoing monitoring of telehealth access and use across sociodemographic groups is necessary to identify persistent disparities. Analyzing telehealth use by race or ethnicity, age, rurality, and socioeconomic indicators enables stakeholders to detect and respond to inequities.

In addition, clinician preparedness and effective workflow integration are essential for sustaining telehealth services. Training health care providers to deliver equitable and culturally sensitive virtual care, supported by standardized workflows and integrated language interpretation services, may improve clinical quality and patient satisfaction [31,32,36].

### Strengths and Limitations

One of the strengths of this study is the use of comprehensive health care use data from the Virginia APCD, which allowed a detailed analysis of temporal variation in telehealth use across multiple years. The application of Bayesian spatial modeling further strengthened the analysis by accounting for spatial dependencies and providing robust estimates of the associations between telehealth use and community-level factors. However, this study is not without limitations. The observational nature of the analysis limits the ability to draw causal inferences about the relationships between telehealth use and the independent variables. Thus, the findings may not be generalizable to other population groups or settings. In addition, the reliance on claims data may introduce misclassification bias, as not all telehealth visits may be accurately captured in the APCD. Furthermore, this study did not differentiate between video and audio-only telehealth encounters, which is an important consideration, as audio-only visits may help reduce disparities by addressing barriers, such as limited broadband access and digital literacy challenges.

### Future Research Directions

Future research should focus on longitudinal studies to monitor post–COVID-19 pandemic telehealth trends and assess the long-term impact of telehealth on health care access and outcomes. Qualitative studies exploring the barriers to telehealth adoption, such as digital and telehealth literacy, technological access, and perceptions of care quality, would provide valuable insights into the challenges faced by underserved populations. Finally, intervention studies that test the effectiveness of targeted strategies to increase the telehealth adoption in underused areas would contribute to the development of more equitable health care delivery models.

### Conclusions

This study provides a comprehensive understanding of telehealth use trends and disparities in Virginia during the COVID-19 pandemic. The findings highlight the significant increase in telehealth adoption during the COVID-19 pandemic as well as the persistent disparities in access among certain populations and areas. To ensure equitable telehealth delivery, targeted interventions, investments in broadband infrastructure, user-friendly digital platforms, and supportive policies are essential to ensure that telehealth remains a viable and equitable mode of health care delivery in the post–COVID-19 pandemic era.

## Abbreviations

APCD: All-Payer Claims Database
ACS: American Community Survey
DIC: deviance information criterion
IRB: institutional review board
RR: relative risk
SDI: Social Deprivation Index
ZCTA: zip code tabulation area

## Data Availability

The datasets generated or analyzed during this study are not publicly available due to ethical restrictions and patient confidentiality but are available from the corresponding author on reasonable request.
